# A Randomized Controlled Trial of Whole Body Vibration Exposure on Markers of Bone Turnover in Postmenopausal Women

**DOI:** 10.4061/2011/710387

**Published:** 2011-06-27

**Authors:** Sarah Turner, Margaret Torode, Mike Climstein, Geraldine Naughton, David Greene, Michael K. Baker, Maria A. Fiatarone Singh

**Affiliations:** ^1^Exercise, Health and Performance Faculty Research Group, Faculty of Health Sciences, The University of Sydney, Lidcombe, NSW 1825, Australia; ^2^School of Exercise Science, Australian Catholic University, Strathfield, NSW 2135, Australia; ^3^Centre of Physical Activity Across the Lifespan, Australian Catholic University, Fitzroy, Victoria 3065, Australia; ^4^School of Exercise, Biomedical and Health Sciences, Edith Cowan University, Joondalup, WA 6027, Australia; ^5^The Boden Institute of Obesity, Nutrition, and Exercise & Eating Disorders, Sydney Medical School, The University of Sydney, Sydney, NSW 2006, Australia; ^6^Sydney Medical School, The University of Sydney, Sydney, NSW 2006, Australia; ^7^Hebrew SeniorLife and Jean Mayer USDA Human Nutrition Center on Aging, Tufts University, Boston, MA 02111-1524, USA

## Abstract

*Purpose*. To examine the effects of two doses of low-frequency (12 Hz), low-magnitude (0.3 g), whole body vibration on markers of bone formation and resorption in postmenopausal women. *Methods*. Women were recruited and randomized into a sham vibration control group, one time per week vibration group (1×/week), or three times per week vibration group (3×/week). Vibration exposure consisted of 20 minutes of intermittent vibration for the 1×/week and 3×/week groups, and sham vibration (<0.1 g) for the control group for eight weeks. Double-blinded primary outcome measures were urine markers of bone resorption: N-telopeptide X normalised to creatinine (NTx/Cr) and bone formation: bone-specific alkaline phosphatase (ALP). *Results*. Forty-six women (59.8 ± 6.2 years, median 7.3 years since menopause) were enrolled. NTx/Cr was significantly reduced (34.6%) in the 3×/wk vibration group but not in the 1×/wk vibration group compared with sham control (*P* < .01) group. No effect of time or group allocation was observed on the bone formation marker ALP (*P* = .27). *Conclusion*. We have shown for the first time that low-frequency, low-magnitude vibration 3×/week for eight weeks in postmenopausal women results in a significant reduction in NTx/Cr, a marker of bone resorption, when compared with sham vibration exposure.

## 1. Introduction

Whole body vibration training is promoted as a potentially safe, low-impact alternative to current therapeutic modalities to combat bone loss in older adults. Whole body vibration is without the potential side effects of pharmacological intervention or risks associated with high impact or strenuous exercise. The mechanism of vibration stimulus on bone is not wholly understood; however, it is hypothesized that the anabolic effect of vibration on bone may be a result of stress exerted on bone resulting in increased fluid flow [1], greater activation of muscle spindles through enhanced sensitivity of mechanoreceptors, and increased osteogenesis in osteoblasts, suggesting that vibration is anabolic at a cellular level [2, 3].

Animal studies investigating the effects of vibration stimulus on bone reported positive results, with improvements in bone strength, formation rates, bone mineral content, and bone mineral density (BMD) following low-magnitude, high-frequency vibration exposure [4–8]. Human studies have reported potential benefits in bone [9–18], muscle function [15, 17, 19, 20], balance and prevention of falls [21], reduction of muscle spasticity in those with cerebral palsy [22], and postural control in those with Parkinson's disease [23]. However, the optimal time course, dose, and frequency of vibration to elicit optimal changes in bone are currently not established, with a wide variety of vibration exposure protocols leading to changes in bone outcomes in only a small number of the currently published studies. Inconsistency in study nomenclature, design, including measurement of bone outcomes, reporting of adverse effects, and noncompliance, highlight the lack of uniformity and small pool of information available on the effects of vibration on bone [24]. As a result, more robust studies are required in order to understand the effect of vibration on bone and determine the vibration prescription to elicit the greatest beneficial effect on bone.

In this study, we specifically examined the dose-response relationship (frequency of exposure) of vibration stimulus on markers of bone turnover in postmenopausal women in a community setting. We hypothesized the application of low-frequency, low-magnitude vibration stimulus for 8 weeks would increase the bone formation marker, ALP, and decrease the bone resorption marker, NTx/Cr, in a dose-dependent relationship compared with a sham vibration exposure.

## 2. Methods

### 2.1. Participants

Postmenopausal women were recruited from northern suburbs of Sydney, Australia by means of advertisements in local newspapers, posters in local GPs surgeries, letterbox drops, and advertisements to existing members of a large suburban community club via newsletters and posters within the club. 

Participants were considered eligible if they were postmenopausal for at least 12 months; willing to continue taking any bone altering medications or supplements they were previously taking for the duration of the study, including calcium, vitamin D, bisphosphonates, and hormone replacement therapy (HRT); able to stand unassisted for sustained periods of time (i.e., 20 minutes); willing to attend testing and training sessions as determined by the researchers; able to travel independently to and from the testing/training venue. Exclusion criteria included cognitive impairment, contraindications to vibration platform training (including pacemaker and fracture within the past six months), and the diagnosis of diseases other than osteoporosis affecting bone. The study protocol was approved by the Human Research Committee, University of Sydney, Australia (June 2007) and was registered with the Australian Clinical Trials Registry (ACTRN12607000491460). All participants gave informed written consent prior to enrolment.

### 2.2. Design

The study was a randomized placebo-controlled clinical trial, with a complete case statistical analysis design and secondary intention-to-treat analytic strategy. The vibration protocol consisted of an 8-week whole body vibration exposure program. Serum and urine markers of bone turnover were measured at baseline and at the conclusion of the study. All testing and training was conducted at Freshwater Health and Fitness, Harbord Diggers-Mounties Group, Harbord, NSW, Australia. Participants were recruited and trained over one season (late autumn to winter 2007).

### 2.3. Randomization Method, Concealment, Allocation, and Implementation

Participants were randomized into the Sham group, 1×/week group, or 3×/week group following completion of baseline assessments. Randomization was performed offsite by a researcher who was not involved in testing and training of participants using computer-generated randomly permuted blocks (http://www.randomization.com/) in blocks of six. Participants were stratified by years since menopause (<5 years or ≥5 years rounded to the nearest year), use of bone altering medication/supplements for the prevention, and/or treatment of osteoporosis and habitual physical activity over the past 3 months (y/n) defined as at least 2 sessions/week of moderate-to high-intensity sport or recreational activity other than casual walking. Participants were informed of their group allocation by means of sealed opaque envelopes given to them after completion of all baseline testing, which they opened themselves.

### 2.4. Vibration Exposure

Participants who were asked to remain in a standing position on a whole body vibration device. The purpose built device produced a synchronous vibration resulting in vertical acceleration, engineered by Australian Catholic University. Low-frequency, low-magnitude vibration was applied (12 Hz, 0.5 mm peak-to-peak displacement, 0.3 g), consistent with current research that suggests that low magnitude is anabolic to bone [11] as it does not cause damage to physiology associated with high-magnitude vibration [25]. Past literature has reported that low-frequency vibration (<25 Hz) has greatest transmission up the axial skeleton, with this transmissibility decreasing as higher vibration frequencies are utilized [26, 27]. All participants were instructed to stand on the vibration platform with their feet shoulder width apart, knees locked, and hands by their side to receive maximum vibration exposure, as determined in a pilot study performed prior to and during our study period (data not shown). The protocol was performed with participants' shoes removed to prevent any attenuation of vibration that may result from footwear. Participants were blinded to which groups were sham and active. The sham platforms transmitted minimal vibration (<0.1 g) but produced the same audible noise as the active platforms. All of the vibration platforms were covered with fabric to prevent participants from visually identifying active or static platforms. Participants were told that any of the three groups may experience improvements in the outcomes measured and were not aware of the investigators hypotheses regarding frequency of exposure effects.

Participants in the 1×/week and 3×/week groups were required to attend sessions either once or three times a week over eight weeks for 20 minutes of intermittent vibration, respectively, during which time, vibration was applied for one minute, followed by one minute of rest. The osteogenic effect of vibration is reported to be greater when rest breaks are incorporated into the vibration stimulus, as intermittent exposure provides osteoblasts and mechanoreceptors of bone at rest from vibration stimulus, preventing insensitivity to vibration that can occur during prolonged vibration exposure [28]. The sham group attended training once a week and received 20 minutes of a continuous sham vibration. Make-up sessions were offered over a four-week period following the initial 8-week program and thereby provided a 12-week window in order to complete 100% of the prescribed sessions.

### 2.5. Outcomes

Outcome measures were conducted at baseline (before randomization) and at followup (completion of 8 sessions for Sham and 1×/week groups and 24 sessions for 3×/week group).

All outcomes were blindly assessed and analyzed at baseline, as assessment occurred prior to randomization. Outcomes assessed included anthropometric measures, demographic characteristics (including socioeconomic status, smoking habits, and caffeine consumption), habitual physical activity, 25-OH vitamin D status, and body composition measurement by bioelectric impedance analysis (fat-free mass (FFM), skeletal muscle mass (SMM), fat mass (FM)) using standard equations for older adults [29]. 

#### 2.5.1. Primary Outcomes

Markers of bone formation (bone-specific alkaline phosphatase (ALP)) and resorption (N-telopeptide X/Creatinine (NTx/Cr)) were assessed via blood and urine tests performed without batching by an independent laboratory that was blinded to group allocation. Blood samples were collected between 72 and 120 hours following the last vibration exposure session to standardize previously reported acute bout effects on markers of bone metabolism [30].

#### 2.5.2. Compliance and Adverse Events

Compliance for each participant was calculated as a percentage of the prescribed sessions attended. Throughout the eight week study period, a weekly questionnaire was completed in person or via phone calls, in order to monitoring possible adverse effects from the vibration exposure and any changes in health status.

#### 2.5.3. Sample Size Calculations

Based on results from previous research investigating the anabolic effects of exercise on bone markers [31] using an average estimate of difference in bone-specific alkaline phosphatase (ALP) between control and vibration-exposed participants of 2.9% (ES = −1.01), a sample size of 13 per group was required for each of the contrasting dose-response comparisons. This provided a power of 0.80 at an alpha level of 0.05. 

### 2.6. Statistical Analysis

Statistical analyses were performed using StatView statistical software package (Version 5.0 SAS Institute, Cary, NC). Data distributions were inspected for normality. Normally distributed data were described using mean ± SD and non-normally distributed data using median and ranges. Complete case analyses compared the differences in primary and secondary outcomes between the 3×/week, 1×/week, and Sham control group using all available data independent of compliance level. A secondary sensitivity analysis was performed using an intention-to-treat analysis with conservative imputation of results via last value carried forward method. Analysis of covariance (ANCOVA) models were constructed to compare 3×/week, 1×/week, and sham control groups, using the percentage change scores (posttest − pretest/pretest × 100) as the dependent variable, adjusted for baseline values of NTx/Cr and ALP. Variables that were potentially related to the outcome of interest such as age, month of assessment, percent change scores for fat-free mass (FFM), skeletal muscle mass (SMM), body weight, vitamin D, habitual physical activity, use of HRT, and height at baseline were all identified a priori as potential confounders of the treatment effect that could be used as covariates in analysis of covariance (ANCOVA) models of change scores. Variables with clinically importance between group differences were included in ANCOVA models. Post-hoc least significant difference (LSD) *t*-tests were used for all pair-wise comparisons where appropriate (*P* < .05) to ascertain specific group mean differences. Relationships between continuous variables as potential predictors of change in primary outcomes were analyzed with simple and forward stepwise multiple linear regression models. A *P* value of <.05 and/or 95% confidence interval (CI) exclusive of zero were accepted as statistically significant. Effect sizes were judged as small, moderate, and large according to the conventions of Cohen [32]. Clinical relevance was assessed relative to the meaningfulness of bone and musculoskeletal outcomes compared with existing literature.

## 3. Results

**Figure 1:**
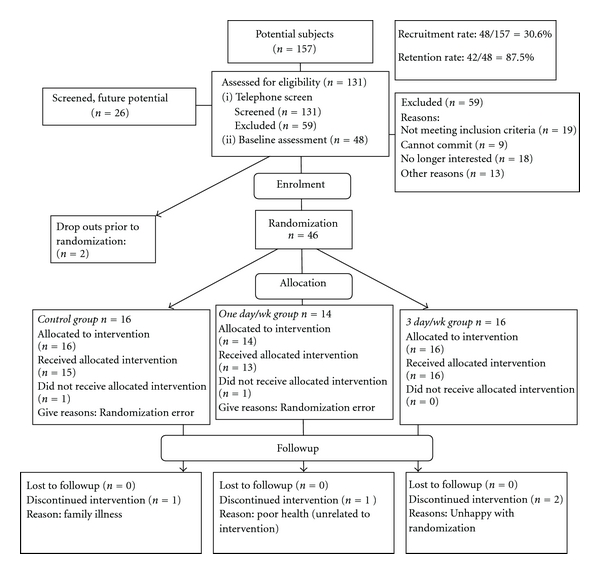
Recruitment flowchart.

Recruitment and enrolment of 46 participants occurred from May to July, 2007. Participant flow through the study is presented in Figure 1.

### 3.1. Participant Characteristics

Baseline participant characteristics are presented in Table 1. The cohort had a mean age of 59.8 ± 6.2 years, with a median 7.3 years since menopause. Thirty-seven percent of participants were overweight, and 4.4% participants were obese, with the majority of participants having BMIs within the normal range. No participants were classified as underweight [33]. Baseline characteristics of the participants did not differ between groups, except for height with participants in the sham group shorter than the rest of the cohort. Osteoporosis, osteoporotic fractures, or osteopenia were present in 30% of the total cohort, with osteopenia diagnosed in 15% of all participants. Osteoarthritis was the highest reported comorbidity in this cohort (41%), with participants also reporting chronic musculoskeletal pain (22%) and hypercholesterolemia (13%) prior to commencement of the study. 

Vitamin D status varied across the cohort, with the majority of participants within the normal range (80%); however, 20% were either classified as mildly or moderately deficient. Following the 8-week study period, Vitamin D levels decreased in all groups, with only 68% of participants classified as normal and an increase in the number of women who were mildly or moderately deficient (33%). This pattern is consistent with expected seasonal change, in which vitamin D levels tend to decrease over winter months [34]. 

The NTx/Cr levels were not different between women stratified by years since menopause (*P* = .92), vitamin D category (normal, mildly, or moderately deficient) (*P* = .42), and participation in regular physical activity (*P* = .95). However, NTx/Cr was lower in women taking medications that affected bone metabolism, consistent with lower levels of bone resorption in these women (*P* = .03). Levels of NTx/Cr were lower in women who were taking calcium supplements (*P* = .01), bisphosphonates (*P* < .01), and HRT (*P* = .09), compared with those not on these agents, but vitamin D supplementation did not appear to influence bone resorption (*P* = .58). Levels of NTx/Cr were higher in participants without a diagnosis of osteoporosis, osteopenia or osteoporotic fracture, who were not taking the above bone-active agents (*P* = .03).

### 3.2. Outcomes

#### 3.2.1. Bone Resorption

**Figure 2:**
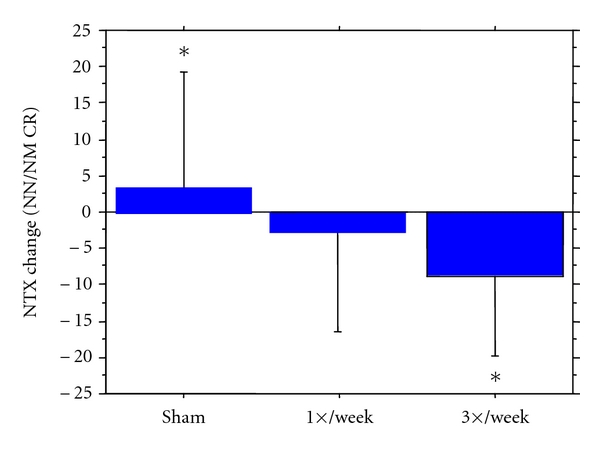
Group effect: Absolute changes in NTx/Cr (nm/mmCr) values presented as Mean ± SD. Analysis by ANCOVA model with changes score as dependent variable and group assignment, baseline value of Ntx/Cr, age, height, use of HRT, and years since menopause as independent variables. Group effect for model was *P* < .05, post-hoc pair-wise comparisons analyzed by post-hoc LSD *t*-tests. *Indicates significance (*P* < .018) between the Sham and 3×/week group.

Following eight weeks of vibration exposure, NTx/Cr levels changed differentially among the three groups. Specifically, NTx/Cr decreased in the 1×/week and 3×/week groups and increased in the sham group, indicating a significant effect of group allocation on bone resorption when compared by repeated measures ANOVA (*P* < .03). Results are presented in Table 2. The change in NTx/Cr was associated with baseline NTx/Cr values but not with any other participant characteristics at baseline or with changes in any participant characteristics. When baseline differences in age, height, NTx/Cr values, use of HRT, and years since menopause were included as covariates in an ANCOVA model (Figure 2), the change in NTx/Cr was different between the 3×/week and sham groups with a net decrease in the 3×/week group of 34.6% (mean difference −12.6 nm/mm Cr, *P* < .01). The change in NTx/Cr did not differ significantly between the active platform groups (*P* = .21) or between the 1×/week group and sham group (*P* = .12).

#### 3.2.2. Bone Formation

The bone formation marker, ALP, tended to decrease in all groups over the eight-week period (*P* = .08), and no group effect on this change was observed (*P* = .27) when compared by repeated measures ANOVA (Table 2). The group effect remained nonsignificant following adjustment for age, height, baseline NTx values, use of HRT, and years since menopause in ANCOVA models. Changes in bone formation were not associated with participant characteristics at baseline or by changes in any participant characteristics. 

#### 3.2.3. Secondary Analyses

Due to low numbers not handled by stratification, the number of participants taking bisphosphonates and on HRT therapy were uneven between groups. In order to correct for any bias due to the potential confounding effect of bisphosphonate and HRT use on bone metabolism, secondary analysis was conducted excluding those participants (*n* = 10). In this analysis, NTx/Cr levels did not change differentially among the three groups when compared by repeated measures ANOVA. When change scores were analyzed using an ANCOVA model, adjusting for baseline differences in age, height, NTx/Cr values, and years since menopause as covariates, the change in NTx/Cr remained different between the 3×/week and sham groups with a net decrease in the 3x/week group of 32.1% (*P* < .03). The change in NTx/Cr did not differ significantly between the active platform groups (*P* = .22) or between the 1×/week group and sham group (*P* = .33). Data were missing for NTx and ALP in 4 (8.7%) participants, due to dropout. A secondary sensitivity analysis using an intention-to-treat model with imputation of data failed to yield any significant groups effects on markers of bone resorption and formation (data not shown).

#### 3.2.4. Compliance

In the three study groups, the reported median vibration training compliance was 100 (0–100%), with 87.5% of randomized participants completing the study. One participant in the sham group withdrew from the study due to family illness, one participant in the 1×/week group withdrew due to illness and family problems, and two participants in the 3×/week group withdrew prior to their first vibration exposure session due to inability to commit to study requirements.

#### 3.2.5. Adverse Events

Adverse effects were reported by five participants over the course of the study. Three participants reported dizziness while standing on the vibration platform (sham,  *n* = 2; 3×/week, *n* = 1); one subject reported feeling faint and was unable to complete 20 minutes of vibration (1×/week,  *n* = 1). One subject reported shoulder pain following baseline muscle strength testing, which persisted for the duration of the study (1×/week, *n* = 1).

## 4. Discussion

In this study, we investigated the frequency dose-response relationship of whole body vibration stimulus on markers of bone turnover in postmenopausal women in a community setting. We showed, for the first time, that low-magnitude, low-frequency vibration exposure (0.3 g) 3 times per week reduced a marker of bone resorption (NTx/Cr) in postmenopausal women. The net decrease in NTx/Cr in the 3×/week group compared with the sham group was 34.6%, which was not only statistically significant, but also potentially *clinically meaningful*. The magnitude of effect remained large (32.1%) when participants taking bisphosphonates and on HRT were removed from the analysis. By comparison, reductions of 25% in markers of bone resorption have been reported in postmenopausal women with osteoporosis/osteopenia following walking programs of 12-month duration [35] and long-term pharmacological treatment [36]. We did not observe an effect of vibration exposure at either frequency on bone formation as assessed by ALP. 

### 4.1. Bone Resorption Markers

Only one previous study by Iwamoto et al. has investigated the effects of vibration on urinary NTx, reporting a nonsignificant decrease following high frequency, low-magnitude vibration exposure over 12 months [12]. The magnitude of vibration in Iwamoto's study was higher than in ours; however, the exposure was only 4 minutes 1×/week and their study investigated additional effects of vibration in participants who were also taking Alendronate, a bisphosphonate with strong bone protective effects of reducing bone resorption by up to 5% following long-term use [36]. A lack of comparison between a vibration group and a control group in the absence of alendronate administration makes it difficult to determine whether there was any isolated effect of low-magnitude vibration on bone resorption. Two studies have investigated other markers of bone resorption (serum C-telopeptide X [16, 17] and Tartrate-resistant acid phosphatase-5b [16]), without significant effects of vibration after 6–8 months of exposure. Thus, the present study is the first to report significant changes in bone resorption following low-magnitude vibration exposure.

Our study suggests a dose-response relationship between vibration exposure and reduced bone resorption. Specifically, vibration exposure 3×/week significantly reduced NTx/Cr when compared with the sham group, but this difference was not seen in the 1×/week group. A large effect size suggests that sample size was adequate to highlight group differences between the 3×/week and sham group (ES = −0.96). However, a small effect size was obtained when comparing the 1×/week group to the sham group, with low statistical power a potential factor in this lack of significance. Larger studies are needed to confirm this lack of efficacy of once-weekly training. However, it remains unknown whether vibration stimulus 3×/week is optimal or whether similar or greater changes in NTx/Cr could be achieved with higher exposure to vibration (number of days per week or minutes per session).

### 4.2. Bone Formation Markers

The bone formation marker ALP decreased similarly in all groups over the study period, which may be explained by the late autumn to winter period of recruitment into the study [37]. No studies have investigated the effects of vibration on ALP, but nonsignificant reductions in osteocalcin, another marker of bone formation [16, 17], have been reported. It is also possible that osteocalcin, a more specific marker of bone formation than ALP, is more sensitive to changes in bone formation resulting from vibration exposure. A nonsignificant decrease in osteocalcin following 6 months of high-frequency, high-amplitude vibration has been reported in postmenopausal women [17]. The effect size for this change was large (ES = −0.95), suggesting that the study was adequately powered to identify group differences between the vibration and control groups. This is the only study in humans that has shown a potential negative effect of vibration on markers of bone formation. However, this result is consistent with reports by Rubin indicating that high-amplitude vibration may be catabolic to bone [26]. A decrease in bone mineral density was not found by Verschueren et al., but this may be due to the short duration of the study (6 months). Thus, further research is required to determine if this potential catabolic effect of high amplitude vibration on bone is consistent and related to duration of exposure. 

The lack of improvement in bone formation markers observed in our study is consistent with previous research in older adults. The effect size for changes in ALP between groups in our study were small, suggesting that a larger sample size was required to find a group effect, if one existed, on bone formation levels. The significant reduction in the bone resorption marker we observed is a novel finding after vibration exposure in humans. Notably, the biological plausibility of this finding is supported by a recent study of murine osteocytes in which low-magnitude (0.3 g), high-frequency vibration similar to our protocol increased soluble inhibitors of osteoclast formation at both the transcript and protein level. Resorption activity of the exposed osteocytes was also lowered in response to this vibration stimulus [38].

Large consumption of caffeine is associated with increased risk of osteoporosis and decreased bone formation [39]. This relationship was not found in this cohort with bone formation being similar in participants who consumed multiple cups of caffeinated drinks per day and those who refrained from caffeine consumption.

Compliance for traditional treatments for osteoporosis, including pharmacological aids, have been shown to be low in previous studies [40], and this may be attributed to risks associated with prolonged use of some treatments, such as increased risk of breast and uterine cancers [41]. Compliance in our study was high across all three groups (median 100%; range 0–100%), which is consistent with compliance found in other studies [42], highlighting the potential feasibility of vibration exposure in postmenopausal, community-dwelling women. 

Limitations to this study include sample size and intervention. The intervention period of eight weeks may not have been sufficient to observe larger effects on markers of bone metabolism. Previous studies have shown changes in bone outcomes typically occur over longer intervention periods, especially if architectural changes in bone are to be observed [9–11, 15–17]. Optimal dose and duration of vibration exposure remains unknown, and it is recommended that additional studies be conducted. Our secondary, unplanned analysis with bisphosphonate and HRT users excluded did not show a significant effect of vibration on resorption using repeated measures ANOVA. This is likely due to type II error as the magnitude of effect is similar with these participants excluded. As, by design, the dosages of bone altering medications remained constant throughout the study constant, our results suggest that the observed effect is due to the vibration exposure, rather than an effect of the medication.

Vibration exposure protocols have varied widely in all studies to date investigating the effect of vibration on bone outcomes. While our study compared two vibration intervention groups differing in the number of sessions per week, no human studies have directly investigated if any differences exist in bone outcomes between continuous and intermittent vibration or if a difference exists in the effect of vibration on bone in participants standing still on the platform compared with participants performing dynamic and static exercise while on the platform. Another underexplored aspect of the research is the direction of vibration that elicits the greatest gains in bone outcomes, that is, reciprocal or oscillating. At present, no standard approach exists to counteract the attenuation effects of wearing footwear while on the vibration platform and differences between receiving vibration exposure while wearing shoes or while barefoot, as well as with locked, soft, or flexed knee positions should be investigated. Future research needs to establish whether vibration exposure effects on bone are retained after the vibration stimulus is removed, and the time course of detraining. It is unknown whether gender, nutritional status, hormonal status, bone density, or use of medications affecting bone remodeling influences the efficacy of vibration exposure, and studies comparing responses across such cohorts are required. Finally, the molecular mechanisms which may underlie vibration effects on bone turnover are largely unknown.

## 5. Conclusion

Low-frequency, low-magnitude vibration (12 Hz, 0.3 g) three times a week leads to a potentially clinically meaningful 34.6% reduction in NTx/Cr, a marker of bone resorption, whereas one day per week exposure appears insufficient. Further studies are required to extend and confirm these findings, determine the optimal dose of vibration exposure, and determine whether this decreased resorption is sustained and ultimately leads to increased bone density, tensile strength, and reduced risk of fragility fracture. The effect of long-term vibration on bone resorption may be clinically relevant if the changes we observed after 24 sessions were to be sustained or magnified with greater vibration dosage.

## Figures and Tables

**Table 1:** Demographics and health status.

Variable	Whole cohort (*n* = 46)	Sham (*n* = 16)	1×/wk (*n* = 14)	3×/wk (*n* = 16)
Age (yrs)	59.8 ± 6.2	59.8 ± 5.2	60.9 ± 6.5	58.9 ± 7.1
Height (cm)	160.2 ± 5.8	156.9 ± 4.9	162.5 ± 4.6	161.6 ± 6.2
Body weight (kg)	62.80 (51.7–96.7)	62.15 (54.10–71.20)	64.67 (57.20–96.77)	62.88 (51.70–78.57)
Body mass index (kg/m^2^)^§^	24.38 (20.76–37.33)	25.22 (23.191–27.79)	24.64 (20.76–37.33)	23.62 (21.09–32.70)
History of smoking (%)	32.6	28.6	31.3	37.5
Number of medications/day	3.3 ± 2.6	3.2 ± 3.2	3.8 ± 3.3	2.5 ± 3.5
Vitamin D prescription (%)	17.4	12.5	14.3	25.0
Calcium prescription (%)	43.5	50.0	28.6	50.0
Bisphosphonate prescription (%)	13.0	6.3	14.3	18.8
HRT prescription (%)	8.7	0.0	14.3	12.5
Years since menopause	7.25 (2.00–37.00)	9.75 (2.00–30.00)	6.25 (3.50–37.00)	6.50 (2.50–31.00)
Participation in regular structured exercise (%)	71.7	68.8	71.4	75.0
History of osteoporosis, osteopenia, or osteoporotic fracture	30.4	18.8	28.6	43.8

All data presented as mean ± SD for normally distributed data or median (range) for nonnormally distributed data unless otherwise specified.

^§^Body mass index: an indicator of body fat calculated by weight (kg)/Height (m)^2^. Normal values range from 18.5 to 24.9 kg/m^2^. Values ≥25 kg/m^2^ are considered overweight and ≥30 kg/m^2^ obese.

**Table 2:** Primary outcomes.

	Sham (*n* = 15)		1×/week (*n* = 13)		3×/week (*n* = 14)		Repeated measures (*P*)			Effect size (95% CI)	
	Pre	Post	Pre	Post	Pre	Post	Time	Group × time	S versus 1×	S versus 3×	1× versus 3×
ALP (nmol/L)	13.35 (8.3–19.7)	13.20 (7.9–19.1)	14.85 (8.2–33.0)	13.60 (9.40–32.00)	14.01 (7.01–28.00)	13.30 (7.2–24.0)	.08	.27	0.13 (−0.61 to 0.88)	−0.19 (−0.92 to 0.54)	−0.25 (−1.01 to 0.51)
NTx/ creatinine (nm/mm Cr)	42 (13.0–55.0)	41 (18.0–73.0)	38 (21.0–147.0)	42 (22.0–102.0)	42 (24.0– 74.0)	36.5 (8.0–49.0)	.21	.03*	−0.26 (−1.00 to 0.49)	−0.96 (−1.73 to −0.19)	−0.28 (−1.04 to 0.48)

All data presented as median (range).

*Indicates a significant difference between the three groups (*P* ≤ .05).

*P* values are calculated using repeated measures ANOVA to identify significance of any changes over time or group × time interactions.
